# New Hybrid Pyrazole and Imidazopyrazole Antinflammatory Agents Able to Reduce ROS Production in Different Biological Targets

**DOI:** 10.3390/molecules25040899

**Published:** 2020-02-18

**Authors:** Chiara Brullo, Matteo Massa, Federica Rapetti, Silvana Alfei, Maria B. Bertolotto, Fabrizio Montecucco, Maria Grazia Signorello, Olga Bruno

**Affiliations:** 1 Department of Pharmacy, Section of Medicinal Chemistry, University of Genoa, Viale Benedetto XV 3, I-16132 Genova, Italy; matteo_massa@outlook.it (M.M.); federica.rapetti@edu.unige.it (F.R.); obruno@unige.it (O.B.); 2 Department of Pharmacy, Section of Chemistry and Pharmaceutical and Food Technologies, University of Genoa, Viale Cembrano 4, I-16148 Genova, Italy; alfei@difar.unige.it; 3 First Clinic of Internal Medicine, Department of Internal Medicine, and IRCCS Ospedale Policlinico San Martino Genova-Italian Cardiovascular Network, Largo R. Benzi 10, I-16132 Genoa, Italy; Maria.Bianca.Bertolotto@unige.it (M.B.B.); Fabrizio.Montecucco@unige.it (F.M.); 4 Department of Pharmacy, Biochemistry Lab., University of Genoa, Viale Benedetto XV 3, I-16132 Genova, Italy; Mariagrazia.Signorello@unige.it

**Keywords:** pyrazole-4-carbohydrazides, imidazopyrazole-7-carbohydrazides, reactive oxygen production inhibition, platelets, neutrophils, phosphodiesterase inhibitors

## Abstract

Several anti-inflammatory agents based on pyrazole and imidazopyrazole scaffolds and a large library of substituted catechol PDE4D inhibitors were reported by us in the recent past. To obtain new molecules potentially able to act on different targets involved in inflammation onset we designed and synthesized a series of hybrid compounds by linking pyrazole and imidazo-pyrazole scaffolds to differently decorated catechol moieties through an acylhydrazone chain. Some compounds showed antioxidant activity, inhibiting reactive oxygen species (ROS) elevation in neutrophils, and a good inhibition of phosphodiesterases type 4D and, particularly, type 4B, the isoform most involved in inflammation. In addition, most compounds inhibited ROS production also in platelets, confirming their ability to exert an antiinflammatory response by two independent mechanism. Structure–activity relationship (SAR) analyses evidenced that both heterocyclic scaffolds (pyrazole and imidazopyrazole) and the substituted catechol moiety were determinant for the pharmacodynamic properties, even if hybrid molecules bearing to the pyrazole series were more active than the imidazopyrazole ones. In addition, the pivotal role of the catechol substituents has been analyzed. In conclusion the hybridization approach gave a new serie of multitarget antiinflammatory compounds, characterized by a strong antioxidant activity in different biological targets.

## 1. Introduction

Inflammation is a very complex response of mammals’ body to different external injuries, including traumatic events, contact with irritant substances, pathogens invasion and more. There are different stages in inflammation evolution: the acute phase includes the movement of leukocytes from the blood vessel to the tissues and the immune system activation, aiming to damage control. Then, the prolongation of this initially protective response can lead to a state of chronic inflammation that is characterized by the intervention of other type of cells (such as macrophages and lymphocytes) and injured tissues destruction. Several pathologies are caused by chronic inflammation.

Neurodegenerative disorders are typically multi-factorial diseases. Neuroinflammation is one among the different pathological alteration responsible for their onset and, as other multi-target pathologies, is difficult to treat by a mono-therapy. To get around this problem a new medicinal chemistry paradigm suggests the use of multi-targeted compounds, single small molecules able to act with different mechanism of action against two or more target [1,2]. Polypharmacology with multitarget compounds looks like a winner strategy as it retains the advantages of the multi-drugs therapy without the toxicity and compliance problems related to the use of more than one molecule in combination. Since there are different cellular pathways involved in inflammation progression, and also in neuroinflammation, a multi-target molecules could be very useful in several pathologies such as Alzheimer’s disease (AD) or multiple sclerosis (MS).

**Figure 1:**
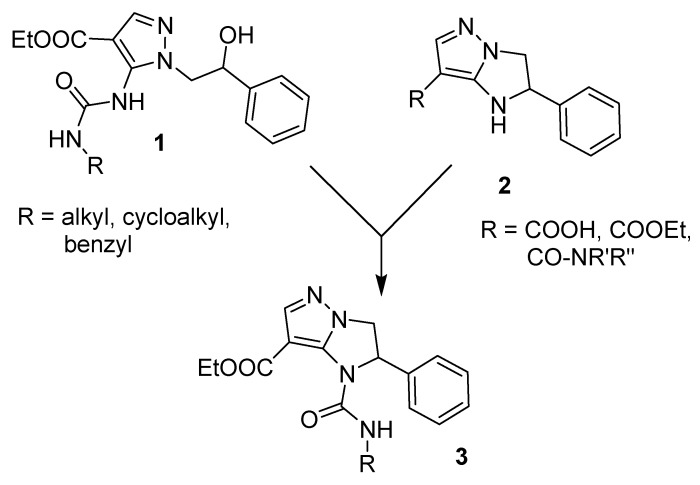
General structure of chemotaxis inhibitors **1**, **2**, **3**.

A multi-target compound can be designed by combining two different molecules (obtaining “chimera” compound) or by overlapping two pharmacophore fragments (obtaining “fused” or “hybrid” molecule). Our previous research on the antiinflammatory agent topic allowed us to identify different pyrazolylureas **1** and imidazopyrazoles **2** (Figure 1) able to inhibit the neutrophil movement towards inflamed tissues (chemotaxis inhibitors) with IC_50_ values in the pico–micromolar range [3,4,5]. Pyrazolylureas **1** inhibited only interleukin 8 (IL8)-induced chemotaxis while imidazopyrazoles **2** inhibited only formylmethyl-leucyl-phenylalanine (fMLP)-induced chemotaxis. The molecular stiffening of previous compounds **1** generates a new class of imidazo-pyrazole derivatives where the urea group is in part inserted in the cycle and in part on the lateral chain (compounds **3**, Figure 1). The new class of compounds **3** obtained by overlapping **1** and **2** was also considered a successful example of hybrid derivatives. Indeed, they showed a good dual activity against both IL-8 and fMLP-induced chemotaxis, with IC_50_ values in the nanomolar range [6], by inhibiting p38 mitogen-activated protein kinase (MAPK) phosphorylation, while no inhibition was observed on superoxide anion production and lysozyme release [7].

**Figure 2:**
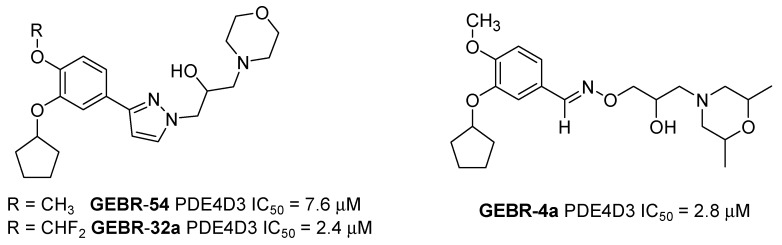
Structures of PDE4D Inhibitors with memory enhancing and anti-inflammatory activities. Corresponding IC_50_ values relative to PDE4D3 inhibition are reported.

In the same years we developed a large library of phosphodiesterase type 4D inhibitors (PDE4DIs). Among them many compounds (particularly **GEBR**-**54** [8] and **GEBR**-**32a** [9], Figure 2) showed interesting PDE4D3 selective inhibition, and in vitro and in vivo assays evidenced their great potential as memory and cognitive performance enhancers (particularly in AD) without causing side-effects, such as sedation or emesis, in comparison with other PDE4Is [8,9,10]. On the other hand, PDE4Is have been largely studied for their anti-inflammatory effects, particularly in autoimmune diseases such as psoriasis [11], asthma and chronic obstructive pulmonary disease (COPD) [12,13]. More recently, several evidences suggested PDE4Is also for neurodegenerative inflammatory diseases [9,14]. The first series of PDE4Is synthesized by our research group showed a great anti-inflammatory activity: in particular, compound **GEBR**-**4a** (Figure 2) and some analogues were able to reduce the superoxide anion and to increase cAMP intracellular level in a dose dependent manner, activity strictly related to PDE4 inhibition [15].

**Figure 3:**
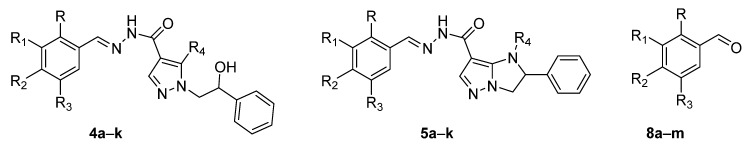
Structures of new hybrid compounds **4a–k, 5a–k** and building block **8a**–**m** used in their preparation.

Supported by the interesting results obtained with hybrid derivatives **3**, and also having in mind the potential role of PDE4Is in inflammation, we designed new hybrid compounds to obtain molecules able to act on inflammation through different mechanism. The pyrazole and imidazo-pyrazole scaffolds present in compounds **1**, **2** and **3** were linked, through an acylhydrazone chain, to a substituted catechol portion that characterized our previous PDE4Is. The acylhydrazone linker was suggested by published analogue PDE4Is [16]. Different alky or aryl substituents were inserted on the catechol oxygens in the new compounds **4** and **5**, to increase the molecular diversity and to obtain more information for structure-activity-relationship (SAR) studies; in many of the new synthesized compounds the OCHF_2_ group has been inserted instead of OCH_3_, in analogy to the more active previous GEBR compounds. Once, also a trifunctionalized phenolic scaffold was used (Figure 3, Table 1). In compound **4k** a pyrrole was inserted in position 5, to verify if a free NH_2_ is necessary for the activity. For the same reason, the NH in position 1 of the imidazopyrazole **5d** was acetylated, thus obtaining compound **5k**.

Compounds **4d** and **5d**, selected on the basis of their similarity with the parental GEBR molecules, were preliminary tested to evaluate the chemotaxis inhibition and H_2_O_2_ production in human neutrophils fMLP stimulated, in order to compare them with compounds **3**. Surprisingly, the compounds showing very low or absent chemotaxis (data not shown), whereas a potent antioxidant activity emerged, as reported in the Results section. 

Inflammation and oxidative stress are deeply connected since inflammation can lead to reactive oxygen species (ROS) production. Oxidative stress occurs in response to injury or to altered metabolic state as a result of an excessive formation of bioactive oxidation products with respect to the capacity of the endogenous scavenging mechanism. ROS are important cellular and tissue damage mediators and are implicated in many pathological processes such as atherosclerosis [17], diabetes [18], neurodegeneration [19], aging [20] and inflammation [17].

As reported before, interesting antioxidant activity was already observed for our PDE4 inhibitor **GEBR-4a** and also other rolipram analogues [21]. Therefore, to clarify if the antioxidant activity revealed by hybrid compounds **4** and **5,** was only due to a cAMP increase or to an independent additional mechanism, we planned to evaluate the ROS production inhibition on human platelets, instead of neutrophils, as platelets are lacking in PDE4 enzymes but, in addition to their central role in the haemostatic process, are involved in the inflammatory process and in the ROS production [22,23].

To date inflammation, thrombosis and haemostasis are reorganized as processes with overlapping steps and connecting pathways. Following injury to blood vessels, such as during inflammation, platelets adhere to endothelium with subsequent thrombin formation. Platelet response to thrombin include cytoskeleton reorganization, degranulation and secretion of proinflammatory cytokines, such as IL-1, tumor growth factor (TGF), platelet derived growth factor (PDGF); therefore thrombin contribute to modifying endothelial function participating in the spreading of an inflammatory lesion [22,23].

## 2. Results and Discussion

### 2.1. Chemistry

**Scheme 1:**
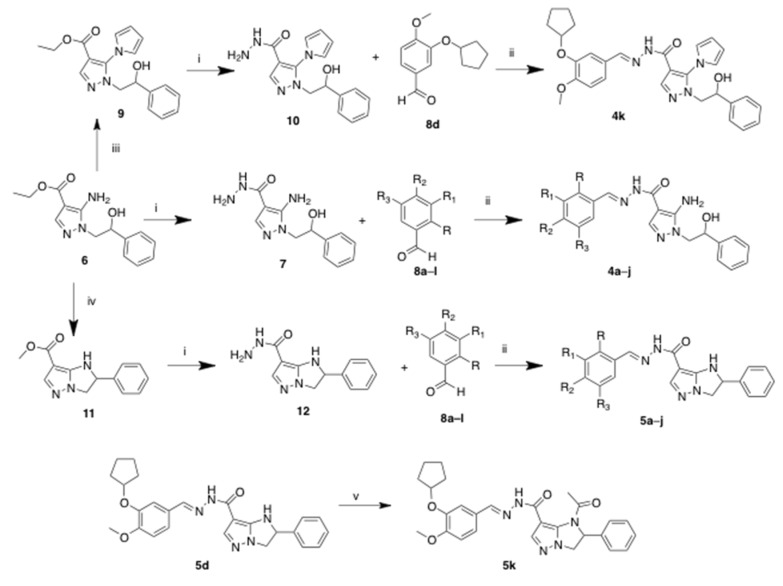
Synthesis of compounds **4** and **5**. *Reagents and conditions:* (**i**) hydrazine monohydrate, 120–130 °C, 4 h; (**ii**) abs. EtOH/MeOH (7:3), reflux, 18 h; (**iii**) 2,5-dimethoxytetrahydrofuran, acetic acid, reflux, 4 h; (**iv**) conc. H_2_SO_4_, 0 °C, 15 min; (v) acetic anhydride, CH_3_COONa, 40–50 °C, 4 h.

Compounds **4a**–**k** and **5a**–**k** (Scheme 1, Table 1) were obtained by refluxing the suitable benzaldehydes **8a**–**m** solved in a mixture of absolute ethanol/methanol (7:3) in the presence of the pyrazolylcarbohydrazides **7, 10** or the imidazopyrazolyl carbohydrazide **12**, respectively.

Although formation of E/Z mixtures is possible, only one of the two isomers has been always obtained as confirmed by ^1^H- and ^13^C-NMR spectra. Since aldehyde hydrazones generally adopt C=N E-geometry [24] and E-geometry was always assigned to the predominant or single isomer obtained [25,26,27,28,29,30], for the prepared acyl hydrazones, the E configuration was a priori hypothesized. Once acquired NMR spectra, the E-geometry was definitively assigned on the basis of the CONH group proton atom chemical shift (δ 11.23 ppm), in accordance to the literature data. In this regard, to a series of hydrazone derivatives, having signals of the NH group <12 ppm as in our case, the E configuration was assigned by NOESY experiments and DFT calculations [31]. Furthermore, it was also reported that NH chemical shift for Z isomers is instead moved to much higher delta values (δ > 15.5 ppm) [32]. ^1^H-NMR spectra images are reported in the Supporting Information.

Intermediate **7** was prepared by reacting an excess of hydrazine monohydrate with the ethyl 5-amino-1-(2-hydroxy-2-phenylethyl)-1*H*-pyrazole-4-carboxylate **6 [33]** (Scheme 1). We obtained the 5-pyrrolyl analogue **9** by refluxing the pyrazolyl ethyl carboxylate **6** in 2,5-dimethoxy-tetrahydrofuran in the presence of acetic acid. Moreover, starting from the same compound **6** we prepared ethyl 2-phenyl-2,3-dihydro-1*H*-imidazo[1,2-*b*]pyrazole-7-carboxylate (**11**) [4] by dehydration in concentrated sulfuric acid. The carbohydrazides **10** and **12** were obtained by the same procedure used for **7**. To prepare derivatives **4** and **5**, the suitable benzaldehydes **8h** and **8m** (Table 1) were purchased, while the others were prepared following well known procedures. In details, the 3-phenoxy-substituted benzaldehydes **8f** and **8i** were obtained by adding diphenyliodonium chloride to 3-hydroxy-4-difluoromethoxy-benzaldehyde [34] (for **8f**) or 2-hydroxy-3-methoxy-benzaldehyde (for **8i**) previously converted into the salt forms with NaOH aqueous solution (Crowder’s method [35]). The others intermediates were prepared by alkylation with the appropriate alkyl chloride in the presence of K_2_CO_3_ [36] starting from the corresponding phenolic benzaldehydes: in particular from 3-hydroxy-4-difluoromethoxy benzaldehyde (to obtain **8a**, **e**, **f**, **g**), from isovanillin (to prepare **8c**, **d**, **l**), from vanillin (for **8k**), and from 2-hydroxy-3-methoxybenzaldehyde (compounds **8b**, **8i**, **8j**). Finally, compound **5k** was obtained from **5d** by treatment with acetic anhydride in the presence of sodium acetate.

### 2.2. ROS Production and Aggregation Inhibition Evaluated in Human Platelets in the Presence of Compounds ***4a–k*** and ***5a–k***

Since in the presence of high intracellular ROS platelets demonstrate increased activation by agonists and ROS elevation leads to platelet aggregation that can in turn worsen the inflammation status, preliminary we have tested the aggregation inhibiting effect of compounds as detailed in the Experimental section. Results of aggregation inhibition and ROS production inhibition are reported in Table 2. Most of the tested compounds were able to inhibit aggregation and ROS production, being **4a**, **4f** and **4g** the most active with IC_50_ values around 10 µM. Interestingly, the same **4a**, **4f** and **4g** were the most active also as platelet aggregation inhibitors.

### 2.3. Structure-Activity Relationships

SAR analysis clearly indicated that the OCHF_2_ substituent at position 4 is a pivotal structural characteristic, while the substituent in position 3 seems not to be determinant for potency. Another clear evidence is that compounds bearing the pyrazole nucleus are more active than the imidazo-pyrazole ones. This is particularly evident by comparing **4b**, **4c**, **4d** and **4e** to the corresponding imidazopyrazole derivatives **5b**, **5c**, **5d** and **5e**, the potency of the first group being approximately from two to four times higher than the second ones.

In addition, we can point out that two substituents in the positions 3 and 4 of the aromatic ring are positive for biological activity, differently than the trisubstitution: in fact, both analogues **4h** and **5h** are inactive. A bulky substituent such as a cyclopentyl in position 2 instead of 3 is well tolerated: indeed, we observed a slight increase of activity in the pyrazole series (compare **4b** with the regioisomer **4d**) or no change in the imidazopyrazole series (compare **5b** with **5d**); whereas, aromatic substituents such benzyloxy or phenoxy in position 2 are detrimental for the activity; in fact, **4i**, **4j**, **5i** and **5j** are completely inactive. A slight reduction of activity is also evident when the meta and para substituents are closed in a ring (see compound **5g**).

Moreover, it is noteworthy that the presence of a free amino group at position 5 of the pyrazole ring is fundamental to block ROS production, as the 5-pyrrolyl compound **4k** showed lower activity compared to the 5-NH_2_-substituted analogue **4d** (IC_50_ = 90 µM versus 40 µM). A similar result was obtained by acetylating the NH group in position 1 of the imidazopyrazole ring, being the acetyl derivative **5k** completely inactive, while the corresponding **5d** showed an IC_50_ value of 70.6 µM. This observation suggests that, even if the imidazopyrazoles are in general less active than the pyrazole ones, the effect on ROS production could be due to the same mechanism, which probably requires an H-bond donor group and a good degree of molecule flexibility.

Finally, we also tested in the same assay the intermediates **7** and **12** in order to confirm that the ROS production inhibition of compounds **4** and **5** is due to the molecule hybridization and not to the pyrazole or imidazopyrazole scaffold alone. As we hypothesized, both **7** and **12** were completely inactive towards ROS production in platelets, as well as in neutrophils (data not shown). For the same reason we tested on platelets also some compounds among the previous series **3** that resulted inactive as well (data not shown).

### 2.4. Flow Cytometric Evaluation of ROS Production on Human Neutrophils in the Presence of Compounds ***4d*** and ***5d***

As reported in the introduction, to verify if the pharmacological activities already evidenced in previous compounds was maintained, preliminary tests were performed on some of the new hybrid compounds (in particular **4d** and **5d**, bearing the catechol structure typical of previously synthesized PDE4DIs). Firstly, the ability of **4d** and **5d** in inhibiting neutrophil chemotaxis were evaluated in order to compare the results with compound **GEBR-4a**. Unexpected negative results (data not shown) suggested that newly synthesized compounds should have a different target. Consequently, the ability to inhibit ROS production was also evaluated on neutrophils fMLP activated using flow cytometric analysis, according to literature method [37]. Interestingly compounds **4d** and **5d** at 10 µM concentration were able to inhibit ROS production with an inhibition percentage of 68% and 63% respectively, using the 2′,7′-dichlorofluorescein diacetate method (DCFH-DA), a fluorogenic dye that measures hydroxyl, peroxyl and other reactive oxygen species. 

**Figure 4:**
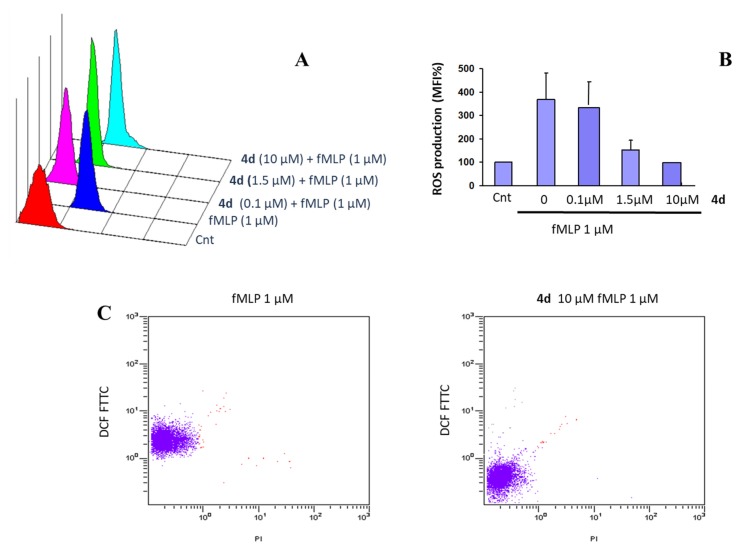
Modulation of neutrophil oxidative metabolism and cell vitality assessed by flow cytometric analysis in the presence of propidium iodide (PI). Representative images relative to evaluation of compound **4d** in formyl-methyl-leucyl-phenylalanine (fMLP)-activated neutrophils. (**A**): reactive oxygen species (ROS) intracellular level reduction (**B**): dose-dependent effect of compound on ROS reduction (**C**): cell vitality determined by PI method. Data are expressed as mean ± SD and are representative of four separate experiments.

In Figure 4 we report the intracellular ROS level inhibition (4A) and the corresponding dose-dependent effect (4B) of **4d** alone, since results were very similar for **5d**. In the same test also the intermediates **7** and **12** were evaluated and resulted inactive (data not shown). In addition, at the same time, using propidium iodide (PI) method [38], we assessed cell vitality and no cytotoxic action of both compounds at 10 µM concentration. Propidium iodide is able to penetrate only the compromised membrane of plasma cells and therefore causes red fluorescence by binding to double-stranded DNA. On the contrary it don’t cross intact plasma cell membrane, therefore no red fluorescence is present in vital cells, as evidenced in Figure 4C. Cell viability was over than 95%.

### 2.5. Enzymatic Assays on PDE4

Compounds **4d** and **5d** were tested on enzymatic assay to verify the ability to inhibit PDE4D3 and PDE4B2, the isoforms most investigated in our previous studies (see experimental section). Results indicated that hybrid compounds **4d** and **5d** are able to strongly inhibit both isoforms and no differences were evidenced between pyrazole or imidazopyrazole scaffold. Indeed, **4d** showed IC_50_ values of 1.05 µM on PDE4D3 and 0.55 µM on PDE4B2; similarly, **5d** showed IC_50_ of 1.22 µM on PDE4D3 and 0.55 µM on PDE4B2. Preliminary evaluation of compound **4d** (at 10 µM concentration) on PDE1, PDE2, PDE3, PDE5 and PDE7 was already performed and no inhibition was observed (data not shown).

## 3. Experimental 

### 3.1. General Information

All chemicals were purchased from Chiminord and Aldrich Chemical (Milan, Italy). Solvents were reagent grade. Unless otherwise stated, all commercial reagents were used without further purification. Aluminium- backed silica gel plates (DC-Alufolien Kieselgel 60 F254, Merck, Darmstadt, Germany), were used in thin-layer chromatography (TLC) for routine monitoring the course of reactions. Detection of spots was made by UV light. Merck silica gel, 230–400 mesh, was used for chromatography. Melting points are not “corrected” and were measured with an M-560 instrument (Buchi instruments, Flawil, Switzerland). IR spectra were recorded with a 398 spectrophotometer (Perkin-Elmer, Milan, Italy). ^1^H NMR spectra were recorded on a Gemini 200 (200 MHz, Varian Gemini, Palo Alto, CA, USA) and a DPX-300 (300 MHz) instrument (Bruker, Billerica, MA, USA); chemical shifts are reported as δ (ppm) relative to tetramethylsilane (TMS) as internal standard; signals were characterized as s (singlet), d (doublet), t (triplet), q (quartet), m (multiplet), br s (broad signal), n t (near triplet); J in Hz. Elemental analyses were determined with an elemental analyzer EA 1110 (Fison-Instruments, Milan, Italy) and the purity of all synthesized compounds was >95% (see Supplementary Materials). Flow cytometric assays were performed by flow cytometry (FC500, Beckman Coulter, Hialeah, FL, USA).

### 3.2. Synthesis

#### 3.2.1. General Procedure for 5-amino-1-(2-hydroxy-2-phenylethyl)-1*H*-pyrazole-4-carbohydrazide (**7**) and 2-phenyl-2,3-dihydro-1*H*-imidazo[1,2-b]pyrazole-7-carbohydrazide (**12**)

A mixture of 5-amino-1-(2-hydroxy-2-phenylethyl)-1*H*-pyrazole-4-carboxylic acid ethyl ester (**6,** 0.81 g, 3.1 mmol) or 2-phenyl-2,3-dihydro-1*H*-imidazo[1,2-*b*]pyrazole-7-carboxylic acid ethyl ester (**11**, 0.8 g, 3.1 mmol) and hydrazine monohydrate (5 mL, 100 mmol) was stirred at 120 °C for 8 h. After cooling to room temperature, water (10 mL) was added and the obtained solids were purified by Florisil column cromatography using a diethyl ether/methanol mixture (9:1) as the eluent. The obtained solids were then recrystallized by ethanol/methanol (2:1).

*5-Amino-1-(2-hydroxy-2-phenylethyl)-1H-pyrazole-4-carbohydrazide* (**7**)**.** White solid. Yield 48%. Mp: 183–184 °C. IR (KBr) cm−1: 3513, 3389 (NH_2_), 3316 (OH), 1603 (C=O). ^1^H-NMR (CDCl_3_): δ 3.90–4.08 (m, 2H, CH_2_N), 4.21 (br s, 2H, NH_2_ disappears with D_2_O), 4.86–5.03 (m, 1H, *CH*OH), 5.72 (d, *J* = 4.6, 1H, OH, disappears with D_2_O), 6.04 (br s, 2H, NH_2_, disappears with D_2_O), 7.21–7.47 (m, 5H, Ar), 7.66 (s, 1H, H_3_ pyraz.), 8.96 (br s, 1H, CONH, disappears with D_2_O). Analysis (%) calcd. for C_12_H_15_N_5_O_2_.

*2-Phenyl-2,3-dihydro-1H-imidazo[1,2-b]pyrazole-7-carbohydrazide* (**12**). White solid. Yield 66%. Mp 250–252 °C. IR (KBr) cm−1: 3296 (NH_2_), 1627 (C=O). ^1^H-NMR (DMSO-d_6_) δ: 3.77 (t, *J* = 8.0, 1H, H_3_), 4.26 (s, 2H, NH_2_, disappears with D_2_O), 4.57 (t, *J* = 8.0, 1H, H_2_), 5.42 (dt, *J* = 4.0, *J* = 8.0, 1H, H_3_), 7.04 (br s, 1H, NH, disappears with D_2_O), 7.26–7.52 (m, 5H, Ar), 7.67 (s, 1H, H_6_), 8.80 (s, 1H, CONH, disappears with D_2_O). Analysis (%) calcd. for C_12_H_13_N_5_O.

#### 3.2.2. Preparation of Ethyl 1-(2-hydroxy-2-phenylethyl)-5-(1*H*-pyrrol-1-yl)-1*H*-pyrazole-4-carboxylate (**9**)

To a solution of 5-amino-1-(2-hydroxy-2-phenylethyl)-1*H*-pyrazole-4-carboxylic acid ethyl ester (**6**) [33] (0.27 g, 1 mmol) in glacial acetic acid (3 mL), 2,5-dimethoxytetrahydrofuran (0.15 g, 1.1 mmol) was added and the mixture was stirred at reflux temperature for 2 h. After cooling to room temperature, the mixture was diluted with water (10 mL) and extracted with ethyl acetate (3 × 10 mL). The organic phase was washed with sodium hydrogen carbonate saturated solution (1 × 10 mL), water (1 × 10 mL), dried (MgSO_4_) and concentrated under reduced pressure to give an oil that was purified by silicagel column chromatography using as the eluent firstly dichloromethane, then diethylether. The product was obtained as a white solid. Yield: 55%. Mp: 103–104 °C. IR (KBr) cm^−1^: 3318 (OH), 1687 (C=O). ^1^H-NMR (CDCl_3_): δ 1.22 (t, *J* = 6.4, 3H, CH_3_), 2.90 (br s, 1H, OH disappears with D_2_O), 4.03–4.29 (m, 4H, CH_2_O + CH_2_N), 5.12–5.22 (m, 1H, *CH*OH), 6.35 (s, 2H, H_2_ pyrr.), 6.58 (s, 2H, H_3_ pyrr.), 7.17–7.52 (m, 5H, Ar), 8.09 (s, 1H, H_3_ pyraz.). Analysis (%) calcd. for C_18_H_19_N_3_O_3_.

#### 3.2.3. Preparation of 1-(2-hydroxy-2-phenylethyl)-5-(1*H*-pyrrol-1-yl)-*1H*-pyrazole-4-carbohydrazide (**10**)

Ethyl 1-(2-hydroxy-2-phenylethyl)-5-(1*H*-pyrrol-1-yl)-1*H*-pyrazole-4-carboxylate (**9**, 0.50 g, 1.53 mmol) was solved in hydrazine monohydrate (5 mL) and the mixture was stirred at reflux for 5 h. After cooling to room temperature, the mixture was diluted with water (10 mL) and extracted with ethyl acetate (3 × 10 mL). The organic phase was washed with brine (2 × 10 mL), dried (MgSO_4_) and concentrated under reduced pressure to give an oil that was purified by Silica gel column chromatography using dichloromethane, as the eluent. The oil residue was crystallized by ethanol obtaining the final product as a white solid. Yield: 61%. Mp: 145–146 °C. ^1^H-NMR (CDCl_3_): δ 3.88 (d, *J* = 6.4, 2H, CH_2_N), 4.29 (br s, 2H, NH_2_ disappears with D_2_O), 4.88-4.99 (m, 1H, *CH*OH), 5.74 (d, *J* = 4.4, 1H, OH disappears with D_2_O), 6.24 (s, 2H, H_2_ pyrr.), 6.78 (s, 2H, H_3_ pyrr.), 7.04–7.41 (m, 5H, Ar), 8.00 (s, 1H, H_3_ pyraz.), 9.07 (br s, 1H, CONH disappears with D_2_O). Analysis calcd. for C_16_H_17_N_5_O_2_.

#### 3.2.4. General Procedure for (*E*)-5-amino-1-(2-hydroxy-2-phenylethyl)-*N*′-benzylidene-1*H*-pyrazole-4-carbohydrazides **4a–k** and *N*′-benzylidene-2-phenyl-2,3-dihydro-1*H*-imidazo[1,2-*b*]pyrazole-7-carbohydrazides **5a–j**

A solution of a suitable benzaldehyde **8** (2 mmol) in a mixture of ethanol/methanol (7:3, 5 mL) was added dropwise to 5-amino-1-(2-hydroxy-2-phenylethyl)-1*H*-pyrazole-4-carbohydrazide (**7**) or 1-(2-hydroxy-2-phenylethyl)-5-(1*H*-pyrrol-1-yl)-1*H*-pyrazole-4-carbohydrazide (**10** )or 2-phenyl-2,3-dihydro-1*H*-imidazo[1,2-*b*]pyrazole-7-carbohydrazide (**12**) (2 mmol) solved in the same mixture solvent (15 mL). The reaction mixture was stirred at 100 °C for 18 h, cooled to room temperature and poured into ice water (10 mL). The white solids obtained were simply dried, if pure at TLC check (Silica gel, diethyl ether/methanol (8:2)), or recrystallized by absolute ethanol or ethyl acetate.

*(E)-5-Amino-N′-(4-(difluoromethoxy)-3-methoxybenzylidene)-1-(2-hydroxy-2-phenylethyl)-1H-pyrazole-4-carbohydrazide* (**4a**). Yield 63%. White solid. Mp: 119–121 °C. ^1^H-NMR (DMSO-d_6_): δ 3.91 (s, 3H, OCH_3_), 3.94–4.15 (m, 2H, CH_2_N), 4.94–5.11 (m, 1H, *CH*OH), 5.72 (s, 1H, OH, disappears with D_2_O), 6.77 (br s, 2H, NH_2_, disappears with D_2_O), 7.10–7.70 (m, 9H, 5Ar + H_2_ Ar + H_5_ Ar + H_6_ Ar + OCHF_2_), 7.82–8.20 (m, 2H, CH=N + H_3_ pyraz.), 11.20 (s, 1H, CONH, disappears with D_2_O). Anal calcd. for C_21_H_21_F_2_N_5_O_4_.

*(E)-5-Amino-N′-(2-(cyclopentyloxy)-3-methoxybenzylidene)-1-(2-hydroxy-2-phenylethyl)-1H-pyrazole-4-carbohydrazide* (**4b**). Yield 46%. White solid. Mp: 180–181 °C. IR (KBr) cm^−1^: 3427 (OH), 3321, 3323, 3139 (NH_2_ + NH), 1634 (C=O), 1572 (C=N). ^1^H-NMR (DMSO-d_6_): δ 1.40–2.00 (m, 8H, 4CH_2_ cyclopent.), 3.78 (s, 3H, OCH_3_), 3.90–4.80 (m, 2H, CH_2_N), 4.80–4.90 (m, 1H, OCH cyclopent), 4.95–5.05 (m, 1H, *CH*OH), 5.65–5.75 (m, 1H, OH, disappears with D_2_O), 6.20–6.50 (br s, 2H, NH_2_, disappears with D_2_O), 6.68 and 7.12 (qAB, *J* =10.0, 2H, Ar), 7.20–7.50 (m, 6H, Ar), 7.80–8.20 (m, 2H, CH=N + H_3_ pyraz.), 11.03 (s, 1H, CONH, disappears with D_2_O). Anal calcd. for C_25_H_29_N_5_O_4_.

*(E)-5-Amino-N′-(3-(sec-butoxy)-4-methoxybenzylidene)-1-(2-hydroxy-2-phenylethyl)-1H-pyrazole-4-carbohydrazide* (**4c**). Yield 64%. White solid. Mp: 190–193 °C. IR (KBr) cm^−1^: 3427 (OH), 3325, 3234, 3196 (NH_2_ + NH), 1644 (C=O), 1614 (C=N). ^1^H-NMR (DMSO-d_6_): δ 0.91–0.94 (m, 3H, *CH_3_*CH_2_), 1.18–1.35 (m, 3H, *CH_3_*CH), 1.40–1.80 (m, 2H, CH_3_*CH_2_*), 3.30–3.40 (m, 1H, *CH*CH_3_), 3.79 (s, 3H, OCH_3_), 3.90–4.20 (m, 2H, CH_2_N), 4.95–5.05 (m, 1H, *CH*OH), 5.60–5.80 (m, 1H, OH, disappears with D_2_O), 6.15–6.55 (m, 2H, NH_2_, disappears with D_2_O), 6.90–7.50 (m, 8H, Ar), 7.70–8.30 (m, 2H, CH=N +H_3_ pyraz.), 11.01 (s, 1H, CONH, disappears with D_2_O). Anal calcd. for C_24_H_29_N_5_O_4_.

*(E)-5-Amino-N′-(3-(cyclopentyloxy)-4-methoxybenzylidene)-1-(2-hydroxy-2-phenylethyl)-1H-pyrazole-4-carbohydrazide* (**4d**). Yield 78%. White solid. Mp: 193–195 °C. IR (KBr) cm^−1^: 3324, 3219 (NH), 2970 (OH), 1615 (C=N), 1642 (CONH). ^1^H-NMR (CDCl_3_): δ 1.48–2.10 (m, 8H, cyclopent.), 3.89 (s, 3H, OCH_3_), 4.02–4.33 (m, 2H, CH_2_N), 4.78–4.99 (m, 1H, OCH cyclopent.), 5.11–5.32 (m, 1H, *CH*OH), 5.82 (br s, 2H, NH_2_ disappears with D_2_O), 6.80–7.01 (m, 1H, H_5_ Ar), 7.04–7.22 (m, 1H, H_6_ Ar), 7.22–7.61 (m, 7 H, 6H Ar + H_2_ Ar), 7.75–8.38 (m, 2H, CH=N + H_3_ pyraz.), 9.00 (br s, 1H, CONH, disappears with D_2_O). Anal calcd. for C_25_H_29_N_5_O_4_.

*(E)-5-Amino-N′-(3-(cyclopentyloxy)-4-(difluoromethoxy)benzylidene)-1-(2-hydroxy-2-phenylethyl)-1H-pyrazole-4-carbohydrazide* (**4e**). Yield 96%. White solid. Mp: 178–180 °C. IR (KBr) cm^−1^: 3450–3200 (NH + OH), 1635 (CONH). ^1^H-NMR (DMSO-d_6_): δ 1.51–2.04 (m, 8H, 4CH_2_ cyclopent.), 3.30–3.40 (m, 2H, CH_2_N), 3.90–4.21 (m, 1H, *CH*OH), 4.84–5.10 (m, 1H, OCH cyclopent.), 5.75 (s, 1H, OH, disappears with D_2_O), 6.42 (br s, 2H, NH_2_, disappears with D_2_O), 7.06–7.60 (m, 9H, 5Ar + H_2_ Ar + H_5_ Ar + H_6_ Ar + OCHF_2_), 7.83–8.35 (m, 2H, CH=N + H_3_ pyraz.), 11.20 (s, 1H, CONH, disappears with D_2_O). Anal calcd. for C_25_H_27_F_2_N_5_O_4_.

*(E)-5-Amino-N′-(4-(difluoromethoxy)-3-phenoxybenzylidene)-1-(2-hydroxy-2-phenylethyl)-1H-pyrazole-4-carbohydrazide* (**4f**). Yield 38%. White solid. Mp: 184–186 °C. ^1^H-NMR (CDCl_3_): δ 3.92–4.26 (m, 2H, CH_2_N), 5.08–5.23 (m, 1H, H_3_ pyraz.), 6.60–6.78 (br s, 2H, NH_2_, disappears with D_2_O), 7.17–7.38 (m, 14H, 10Ar + H_2_ Ar + H_5_ Ar + H_6_ Ar + OCHF_2_), 7.97–8.38 (m, 2H, CH=N + H_3_ pyraz.), 9.18–9.40 (s, 1H, CONH, disappears with D_2_O). Anal calcd. for C_26_H_23_F_2_ N_5_O_4_.

*(E)-5-Amino-N′-(3-(benzyloxy)-4-(difluoromethoxy)benzylidene)-1-(2-hydroxy-2-phenylethyl)-1H-pyrazole-4-carbohydrazide* (**4g**). Yield 61%. White solid. Mp: 144–146 °C. ^1^H-NMR (DMSO-d_6_): δ 3.95–4.20 (m, 2H, CH_2_N), 4.92–5.10 (m, 1H, *CH*OH), 5.26 (s, 2H, CH_2_Bz), 5.75 (s, 1H, OH, disappears with D_2_O), 6.40 (br s, 2H, NH_2_, disappears with D_2_O), 7.16–7.70 (m, 14H, 10Ar + H_2_ Ar + H_5_ Ar + H_6_ Ar + OCHF_2_), 7.83–8.35 (m, 2H, CH=N + H_3_ pyraz.), 11.25 (s, 1H, CONH, disappears with D_2_O). Anal calcd. for C_27_H_25_F_2_N_5_O_4_.

*(E)-5-Amino-1-(2-hydroxy-2-phenylethyl)-N′-(3,4,5-trimethoxybenzylidene)-1H-pyrazole-4-carbohydrazide* (**4h**). Yield 34%. White solid. Mp: 176–178 °C. IR (KBr) cm^−1^: 3490 (OH), 3435, 3391, 3341, 3239 (NH_2_ +NH), 1640 (CO), 1561 (C=N). ^1^H-NMR (DMSO-d_6_): δ 3.36 (s, 3H, OCH_3_), 3.71 (s, 3H, OCH_3_), 3.83 (s, 3H, OCH_3_), 4.40–4.10 (m, 2H, CH_2_N), 4.95–5.05 (m, 1H, *CH*OH), 5.70–5.80 (m, 1H, OH, disappears with D_2_O), 6.25–6.55 (br s, 2H, NH_2_, disappears with D_2_O), 6.98 (s, 2H, Ar), 7.25–7.50 (m, 5H, Ar), 7.80–8.20 (m, 2H, CH=N + H_3_ pyraz.), 11.18 (s, 1H, CONH, disappears with D_2_O). Anal calcd. for C_22_H_24_N_4_O_5_.

*(E)-5-Amino-1-(2-hydroxy-2-phenylethyl)-N′-(3-methoxy-2-phenoxybenzylidene)-1H-pyrazole-4-carbohydrazide* (**4i**). Yield 66%. White solid. Mp: 207–209 °C. IR (KBr) cm^−1^: 3395 (OH), 3324, 3287, 3206, 3239 (NH_2_ +NH), 1646 (CO), 1551 (C=N). ^1^H-NMR (DMSO-d_6_): δ 3.69 (s, 3H, OCH_3_), 3.85–4.15 (m, 2H, CH_2_N), 4.90–5.05 (m, 1H, *CH*OH), 5.69 (d, *J* = 2.0, 1H, OH, disappears with D_2_O), 6.20–6.50 (br s, 2H, NH_2_, disappears with D_2_O), 6.78 (d, *J* = 4.0, 1H, Ar), 7.00 (t, *J* = 4.0, 1H, Ar), 7.15–7.45 (m, 10H, Ar), 7.55 (d, *J* = 4.0, 1H, Ar), 7.60–8.40 (2 m, 2H, CH=N + H_3_ pyraz.), 11.20 (s, 1H, CONH, disappears with D_2_O). Anal calcd. for C_26_H_25_N_5_O_4_.

*(E)-5-Amino-N′-(2-(benzyloxy)-3-methoxybenzylidene)-1-(2-hydroxy-2-phenylethyl)-1H-pyrazole-4-carbohydrazide* (**4j**). Yield 71%. White solid. Mp: 198–200 °C. IR (KBr) cm^−1^: 3415 (OH), 3352, 3151, 3034 (NH_2_ +NH), 1627 (CO), 1564 (C=N). ^1^H-NMR (DMSO-d_6_): δ 3.87 (s, 3H, OCH_3_), 3.90–4.20 (m, 2H, CH_2_N), 4.85–5.10 (m, 1H, *CH*OH), 4.99 (s, 2H, CH_2_Bz), 5.71 (d, *J* = 2.0*,* 1H, OH, disappears with D_2_O), 6.20–6.60 (br s, 2H, NH_2_, disappears with D_2_O), 7.00–7.55 (m, 13H, Ar), 7.80–8.10 (br s, 1H, H_3_ pyraz.), 8.35–8.55 (br s, 1H, CH=N), 11.19 (s, 1H, CONH, disappears with D_2_O). Anal calcd. for C_27_H_27_N_5_O_4_.

*(E)-5-Amino-N′-(3-(cyclopentyloxy)-4-methoxybenzylidene)-1-(2-hydroxy-2-phenylethyl)-1H-pyrazole-4-carbohydrazide* (**4k**). Yield 80%. White solid. Mp: 206–207 °C. IR (KBr) cm^−1^: 3364, 3309 (NH), 2951 (OH), 1598 (C=N), 1656 (CONH). ^1^H-NMR (DMSO-d_6_): δ 1.45–2.15 (m, 8H, cyclopent.), 3.88 (s, 3H, OCH_3_), 3.97–4.19 (m, 2H, CH_2_N), 4.75–4.97 (m, 1H, OCH cyclopent.), 5.17–5.33 (m, 1H, *CH*OH), 6.57 (s, 2H, H_2_ pyrr.), 6.73 (s, 2H, H_3_ pyrr.), 6.78–7.18 (m, 2H, H_5_ + H_6_ Ar), 7.22–7.61 (m, 7H, 5Ar + H_3_ pyraz. + H_2_ Ar), 8.38 (s, 1H, CH=N), 9.85 (br s, 1H, CONH, disappears with D_2_O). Anal calcd. for C_29_H_31_N_5_O_4_.

*(E)-N′-(3,4-Dimethoxybenzylidene)-2-phenyl-2,3-dihydro-1H-imidazo[1,2-b]pyrazole-7-carbohydrazide* (**5a**). Yield 70%. White solid. Mp: 206–207 °C. IR (KBr) cm^−1^: 3432–3213 (NH), 1700–1600 (CONH + C=N). ^1^H-NMR (DMSO-d_6_): δ 3.70–3.92 (m, 7H, 2 OCH_3_ + H_3_), 4.63 (t, *J* = 8.0, 1H, H_2_), 5.48 (n t., 1H, H_3_), 7.02 (d, *J* = 7.8, 1H, H_5_ Ar), 7.09 (dd, *J* = 7.8, *J* = 1.6, 1H, H_6_ Ar), 7.24–7.53 (m, 7H, 5 Ar + H_2_ Ar + H_6_), 7.60–8.40 (m, 2H, CH=N + NH, 1H disappears with D_2_O), 11.10 (s, 1H, CONH, disappears with D_2_O). Anal calcd. for C_21_H_21_N_5_O_3_.

*(E)-N′-(2-(Cyclopentyloxy)-3-methoxybenzylidene)-2-phenyl-2,3-dihydro-1H-imidazo[1,2-b]pyrazole-7-carbohydrazide* (**5b**). Yield 78%. White solid. Mp: 150–151 °C. IR (KBr) cm^−1^: 3392–3234 (NH), 1644 (CONH), 1594 (C=N). ^1^H-NMR (DMSO-d_6_): δ 1.47–2.20 (m, 8H, cyclopent.), 3.72–3.95 (m, 4H, OCH_3_ + H_3_), 4.62 (n t, 1H, H_2_), 4.75–4.92 (m, 1H, OCH), 5.48 (n t, 1H, H_3_), 7.00 (d, *J* = 10.0, 1H, H_5_ Ar), 7.18 (dd, *J* = 10.0, *J* =1.8, 1H, H_6_ Ar), 7.23–7.52 (m, 7H, 5H Ar + H_2_ Ar + H_6_), 7.99 (br s, 2H, NH + CH=N, 1H disappears with D_2_O), 11.07 (s, 1H, CONH, disappears with D_2_O). Anal calcd. for C_25_H_27_N_5_O_3_.

*(E)-N′-(3-(sec-Butoxy)-4-methoxybenzylidene)-2-phenyl-2,3-dihydro-1H-imidazo[1,2-b]pyrazole-7-carbohydrazide* (**5c**). Yield 62%. White solid. Mp: 130–131°C. IR (KBr) cm^−1^: 3373 (NH), 1631 (CONH), 1594 (C=N). ^1^H-NMR (DMSO-d_6_): δ 0.95 (m, 3H, *CH_3_*CH_2_), 1.25 (d, *J* = 5.6, 3H, *CH_3_*CH), 1.45–1.80 (m, 2H, *CH_2_*CH_3_), 3.60–3.95 (m, 4H, OCH_3_ + *CH*CH_3_), 4.20–4.40 (m, 1H, H_3_), 4.60 (t, *J* = 8.0, 1H, H_2_), 5.40 (t, *J* = 8.0, 1H, H_3_), 6.90–7.50 (m, 8H, Ar), 7.95 (br s, 2H, H_6_ + NH, 1H disappears with D_2_O), 11.10 (s, 1H, CONH disappears with D_2_O). Anal calcd. for C_24_H_27_N_5_O_3_.

*(E)-N′-(3-(cyclopentyloxy)-4-methoxybenzylidene)-2-phenyl-2,3-dihydro-1H-imidazo[1,2-b]pyrazole-7-carbohydrazide* (**5d**). Yield 93%. White solid. Mp: 120–124 °C. IR (KBr) cm^−1^: 3415, 3324 (NH), 1581 (C=N), 1633 (CONH). ^1^H-NMR (CDCl_3_): δ 1.47–2.20 (m, 8H, 4CH_2_ cyclopent.), 3.90 (s, 3H, OCH_3_), 4.07 (n t, 1H, H_3_), 4.62 (n t, 1H, H_2_), 4.79–4.95 (m, 1H, OCH cyclopent.), 5.50 (n t, 1H, H_3_), 6.83 (d, *J =* 8.2, 1H, H_5_ Ar), 7.09 (dd, *J =* 8.2*, J =* 1.6, 1H, H_6_ Ar), 7.29 (d, *J =* 1.6, 1H, H_2_ Ar), 7.32–7.52 (m, 5H Ar), 7.82 (s, 1H, H_6_ pyraz.), 8.25 (br s, 1H, NH disappears with D_2_O), 9.18 (s, 1H, CH=N), 9.85 (br s, 1H, CONH disappears with D_2_O). Anal calcd. for C_25_H_27_N_5_O_3_.

*(E)-N′-(3-(cyclopentyloxy)-4-(difluoromethoxy)benzylidene)-2-phenyl-2,3-dihydro-1H-imidazo[1,2-b]-pyrazole-7-carbohydrazide* (**5e**). Yield 62%. Red solid. Mp: 104–105 °C. IR (KBr) cm^−1^: 3228, 2961 (NH), 1642 (CONH), 1599 (C=N). ^1^H-NMR (DMSO-d_6_): δ 1.52–2.00 (m, 8H, 4CH_2_ cyclopent.), 3.82 (n t, 1H, H_3_), 4.64 (n t, 1H, H_2_), 4.87–4.99 (m, 1H, OCH cyclopent.), 5.48 (n t, 1H, H_3_), 7.07 (t, 1H, *J* = 70.0, OCHF_2_), 7.17–7.54 (m, 10H, 8H Ar + H_6_ + NH, 1 H disappears with D_2_O), 7.99 (br s, 1H, CH=N), 11.20 (br s, 1H, CONH, disappears with D_2_O). Anal calcd. for C_25_H_25_F_2_N_5_O_3_.

*(E)-N′-(3-(Cyclohexyloxy)-4-(methoxy)benzylidene)-2-phenyl-2,3-dihydro-1H-imidazo[1,2-b]pyrazole-7-carbohydrazide* (**5f**). Yield 65%. White solid. Mp: 119–120 °C. IR (KBr) cm^−1^: 3584 (NH), 1633 (CONH), 1581 (C=N). ^1^H-NMR (DMSO-d_6_): δ 1.20–2.20 (m, 10H, 5CH_2_ cyclohex.), 3.80–4.10 (m, 4H, OCH_3_ + OCH), 4.25 (n t, 1H, H_3_), 4.52 (n t, 1H, H_2_), 5.39 (n t, 1H, H_3_), 6.60–7.60 (m, 8H, Ar), 7.90 (br s, 1H, CH=N), 8.20 (s, 1H, H_6_), 10.15 (s, 1H, CONH, disappears with D_2_O). Anal calcd. for C_26_H_29_N_5_O_3_.

*(E)-N′-(benzo[d][1,3]dioxol-5-ylmethylene)-2-phenyl-2,3-dihydro-1H-imidazo[1,2-b]pyrazole-7-carbohydrazide* (**5g**). Yield 93%. White solid. Mp: 230–233 °C. IR (KBr) cm^−1^: 3400, 3227 (NH), 1638 (CONH), 1608 (C=N). ^1^H-NMR (DMSO-d_6_): δ 3.82 (n t, 1H, H_3_), 4.62 (n t, 1H, H_2_), 5.43 (n t, 1H, H_2_), 4.96 (br s, 1H, NH disappears with D_2_O), 6.10 (s, 2H, OCH_2_O), 7.00 (d, *J* = 8.0, 1H, H_5_ Ar), 7.14 (dd, *J =* 8.0*, J* = 1.6, 1H, H_6_ Ar), 7.26 (d, *J* =1.6, 1H, H_2_ Ar), 7.32–7.52 (m, 6H, 5H Ar + H_6_), 7.92–8.20 (m, 1H, CH=N), 11.05 (br s, 1H, CONH disappears with D_2_O). Anal calcd. for C_20_H_17_N_5_O.

*(E)-2-Phenyl-N′-(3,4,5-trimethoxybenzylidene)-2,3-dihydro-1H-imidazo[1,2-b]pyrazole-7-carbohydrazide* (**5h**). Yield 74%. White solid. Mp: 120–121 °C. IR (KBr) cm^−1^: 3400–3200 (NH), 1643 (CONH), 1611 (C=N). ^1^H-NMR (DMSO-d_6_): δ 3.60–3.90 (m, 10H, 3OCH_3_ + H_3_), 4.60 (n t, 1H, H_2_), 5.45 (n t, 1H, H_3_), 6.90–7.00 (s, 2H, Ar), 7.18 (s, 1H, H_6_) 7.20–7.50 (m, 5H, Ar), 7.90 (br s, 1H, CH=N), 11.10 (br s, 1H, CONH, disappears with D_2_O). Anal calcd. for C_22_H_23_N_5_O_4_.

*(E)-N′-(2-(Phenoxy)-3-(methoxy)benzylidene)-2-phenyl-2,3-dihydro-1H-imidazo[1,2-b]pyrazole-7-carbo- hydrazide* (**5i**). Yield 81%. White solid. Mp: 265–266 °C. IR (KBr) cm^−1^: 3261, 3060 (NH), 1629 (CONH), 1580 (C=N). ^1^H-NMR (DMSO-d_6_): δ 3.32 (n t, 1 H, H_3_), 3.62–3.82 (m, 3H, OCH_3_), 4.60 (n t, 1H, H_2_), 5.40 (n t, 1H, H_3_), 6.75–7.60 (m, 13H, Ar), 7.86 (br s, 1H, H_6_), 8.28 (br s, 2H, CH=N + NH, 1H disappears with D_2_O), 11.19 (s, 1H, CONH disappears with D_2_O). Anal calcd. for C_26_H_23_N_5_O_3_.

*(E)-N′-(2-(Benzyloxy)-3-(methoxy)benzylidene)-2-phenyl-2,3-dihydro-1H-imidazo[1,2-b]pyrazole-7-carbohydrazide* (**5j**). Yield 78%. Light yellow solid. Mp 200–202 °C. IR (KBr) cm^−1^: 3602, 3173 (NH), 1644 (CONH), 1578 (C=N). ^1^H-NMR (DMSO-d_6_): δ 3.78–3.96 (m, 4H, OCH_3_ + H_3_), 4.61 (n t, 1H, H_2_), 4.99 (s, 2H, CH_2_Bz), 5.44 (n t, 1H, H_3_), 7.00–7.20 and 7.11–7.60 (2 m, 13H, Ar), 7.93 (s, 1H, H_6_ pyraz.), 8.20–8.50 (br s, 2H, CH=N + NH, 1H disappears with D_2_O), 11.23 (s, 1H, CONH, disappears with D_2_O). Anal calcd. for C_27_H_25_N_5_O_3_.

#### 3.2.5. Preparation of (*E*)-1-acetyl-*N*′-(3-(cyclopentyloxy)-4-methoxybenzylidene)-2-phenyl-2,3-dihydro-1*H*-imidazo[1,2-*b*]pyrazole-7-carbohydrazide (**5k**)

A suspension of **5d** (0.45 g, 1 mmol) and sodium acetate (0.13 g, 1 mmol) in acetic anhydride (5 mL) was stirred at 40–50 °C for 4 h. After cooling to room temperature, the reaction was poured into water (20 mL) and extracted with dichloromethane (3 × 10 mL). The organic phase was washed with brine (3 × 10 mL), dried (MgSO_4_) and concentrated under reduced pressure to give an oil that was crystallized by absolute ethanol to give a light yellow solid. Yield: 49%. Mp: 140–141 °C. IR (KBr) cm^−1^: 2959 (NH), 1650 (C=O), 1596 (C=O). ^1^H-NMR (CDCl_3_): δ 1.51–2.20 (m, 8H, 4CH_2_ cyclopent.), 3.91 (s, 3H, OCH_3_), 3.97 (s, 3H, CH_3_CO), 4.15–4.25 (m, 1H, H_3_), 4.78–5.03 (m, 2H, H_2_+ CH cyclopent.), 5.78–5.91 (m, 1H, H_3_), 6.85 (d, *J* = 10.0, 1H, H_5_ Ar), 7.00 (dd, *J* = 10.0*, J* = 2.0, 1H, H_6_ Ar), 7.15–7.25 (m, 1H, H_2_ Ar), 7.35–7.60 (m, 5H, Ar), 8.15 (s, 1H, H_6_), 8.30 (br s, 1H, CH=N), 9.87 (s, 1H, CONH, disappears with D_2_O). Elemental Analysis (%) calculated for C_27_H_29_N_5_O_4_.

### 3.3. Biological Studies

#### 3.3.1. Material and Methods

Compounds **4** and **5** were diluted in saline from a stock DMSO solution immediately before each experiment. 2′,7′-Dichlorofluorescein diacetate (DCFH-DA) and thrombin were purchased from Sigma-Aldrich/Merck Millipore.

#### 3.3.2. Blood Collection and Preparative Procedures

Freshly drawn venous blood from healthy volunteers of the “Centro Trasfusionale, Ospedale San Martino” in Genoa was collected into 130 mM aqueous trisodium citrate anticoagulant solution (9:1). The donors claimed to have not taken drugs known to interfere with platelet function during two weeks prior to blood collection and gave their informed consent. Washed platelets were prepared centrifuging whole blood at 100× *g* for 20 min. The obtained platelet-rich plasma was then centrifuged at 1100× *g* for 15 min. Pellet was washed once with pH 5.2 ACD solution (75 mM trisodium citrate, 42 mM citric acid and 136 mM glucose), centrifuged at 1100× *g* for 15 min and then re-suspended in pH 7.4 Hepes buffer (145 mM NaCl, 5 mM KCl, 1 mM MgSO_4_, 10 mM glucose, 10 mM HEPES).

#### 3.3.3. ROS Assay

ROS production was quantified as previously reported [22,23] by DCFH-DA, a ROS-sensitive probe that yields upon oxidation the fluorescent adduct DCF that is trapped inside the cells. Briefly, washed platelets (1.0 × 10^8^/mL), pre-incubated with saline, compounds **4** or **5**, for 15 min at 37 °C, were stimulated by 0.1 U/mL thrombin. Incubation was stopped by cooling samples in ice bath and then samples were immediately analyzed in a Merck Millipore Bioscience Guava easyCyte flow cytometer (Merk Millipore, Burlington, MA, USA). The reported IC_50_ value is the molar concentration of the compound able to obtain 50% inhibition of the maximal aggregation induced by the agonist and is calculated by the percentage of inhibition that is the inhibition of the maximal aggregation measured in the presence of the agent compared with that measured in a control sample containing saline, carried out under the same conditions.

#### 3.3.4. Platelet Aggregation

Platelet aggregation was performed in a Bio-Data Aggregometer (Bio-Data Corporation, Horsham, PA, USA) according to Born’s method [39] and quantified by the light transmission reached within 6 min at 37 °C. Briefly, washed platelets (3.0 × 10^8^/mL) were pre-incubated for 3 min at 37 °C with saline, compound **4** or **5** before the addition of 0.1 U/mL thrombin. The IC_50_ values were calculated as above detailed.

#### 3.3.5. Isolation of Human Primary Neutrophils

Heparinized (heparin 10 U/mL Vister, PfizerItalia, Borgo San Michele, Latina, Italy) venous blood was obtained from healthy male volunteers after written informed consent. Neutrophils were isolated by dextran sedimentation (Dextran 70.000 Plander, Fresenius Kabi Italia, Verona, Italy), and subsequent centrifugation on a Ficoll-Hypaque density gradient (Lympholyte-I from Cedarlane Laboratories Ltd. Hornby, ON, Canada), as described [37,40]. Contaminating erythrocytes were removed by hypotonic lysis. Neutrophils were washed and resuspended in Hanks’balanced salt solution (HBSS; EuroCLone, Wetherby West, Yorkshire, UK) mixed with Dulbecco’s PBS (EuroClone; HBBS:PBS. Final cell suspensions contained 97% or more neutrophils and more than 98% viable cells, as evaluated by a trypan blue test kit (Sigma Aldrich) [38].

#### 3.3.6. Flow Cytometric Assessment of Neutrophil Oxidative Metabolism

Flow cytometric analysis of neutrophil oxidative metabolism was carried out according to literature method [37]. Briefly, neutrophils, were cultured for 1 h with or without **4d** and **5d** at different concentration. Thereafter, neutrophils were pre-treated with 2′-7′-dichlorofluorescein-diacetate (DCFH-DA; Sigma, Milan, Italy) (5 mM), During the incubation time, DCFH-DA entered into the cells, and it was cleaved by intracellular esterases to give non-fluorescence DCFH trapped within the cells. After washing in PBS, the cells were incubated for 15 min at room temperature in the presence or absence of *N-*formyl-l-methionin-l-leucyl-l-phenylalanine (fMLP; Sigma, Milan, Italy) (1 mM) for 30 min at 37 °C. During this period, intracellular ROS oxidized DCFH to give a green fluorescence DCFH. The reaction was stopped by keeping the samples on ice before being analyzed by flow cytometry (FC500, Beckman Coulter).

In the same test, cell vitality in presence of **4d** (10 µM) was assessed by propidium iodide (PI) method [40,41,42] at the end of the incubation time. In detail, in the same tubes we added PI staining solution (10 μg/mL, Invitrogen ThermoFisher Scientific, Waltham, MA, USA) to detect dead cells. PI is excited at 488 nm and, emits at a maximum wavelength of 617 nm. Because of these spectral characteristics, PI can be used in combination with other fluorochromes excited at 488 nm such as fluorescein isothiocyanate (FITC). Auto-fluorescence levels of sample were measured and subtracted from each analysis. Data were expressed as mean fluorescence intensity ((MFI), compared with the expression on cells stimulated with control medium alone (defined as 100%)).

#### 3.3.7. Enzymatic Assays

PDE4 tests were performed by Scottish Biomedical Drug Discovery (Scottish, Biomedical Drug Discovery, Glasgow, UK) using human recombinant enzyme obtained from *Spodoptera frugiperda* (Sf9) cells using a baculovirus expression system (PDE enzyme at 0.5 U/mL in 20 mM Tris.HCl pH 7.4) as previously reported [8,43]. IMAP technology is based on the high affinity binding of phosphate by immobilized metal coordination complexes on nanoparticles. The binding reagent complexes with phosphate groups on nucleotide monophosphate generated from cyclic nucleotides (10 mM cAMP) through phosphodiesterases. With fluorescence polarisation detection, binding causes a change in the rate of the molecular motion of the phosphate bearing molecule, and results in an increase in the fluorescence polarization value observed for the fluorescent label attached to the substrate. 

Title compounds **4d** and **5d** were solved in DMSO at 10^−2^ M concentration and then diluted with water to the final suitable concentrations. All our subsequent assays were performed in 3% DMSO (final). All the compounds were tested preliminary at 10 µM concentration, in duplicate.

In all the experiments, Rolipram as reference compound (at 10 μM for PDE4B2 and 1 μM for PDE4D3) was tested at nine concentrations, in duplicate, to obtain an inhibition curve in order to validate this experiment.

Being inhibition >50%, **4d** and **5d** have been further tested in the same assay at five different concentrations (5 × 10^−8^–10^−4^ M) and IC_50_ values were determined by nonlinear regression analysis of the inhibition curve, using Hill equation curve fitting (Graph Pad Prism software, version 5.0a).

## 4. Conclusions

Starting from previous anti-inflammatory pyrazole and imidazopyrazole scaffolds and PDE4Is, we designed and synthesized a series of hybrid molecules in order to obtain new antiinflammatory agents able to act on different biological targets. Preliminary tests evidenced a great antioxidant activity in neutrophils for compounds **4d** and **5d**. In addition, the same derivatives showed a good PDE4 inhibition, and also against PDE4B, isoform most involved in inflammation. Results strongly suggested that the PDE4 inhibition is related to antioxidant activity in neutrophils [15]. In addition, the block of ROS production on human platelets, that produce high levels of ROS upon thrombin stimulation by a PDE4-independent mechanism, confirmed that most of tested compounds, except the non-hybrid intermediates, are able to exert an antioxidant response by two independent mechanisms. Interestingly, both heterocycle scaffolds (pyrazole and imidazopyrazole) and the substituted catechol moiety, were determinant for the pharmacodynamic properties. In particular, hybrid molecules bearing to the pyrazole series were more active than the imidazopyrazole ones, confirming that this part is involved in the target interaction. In addition, SAR analyses evidenced a clear relationship between the catechol substituents and the activity.

In conclusion, the hybridization between pyrazole and imidazopyrazole scaffolds and substituted catechol ring through an acylhydrazone linker, gave a new series of multitarget antiinflammatory compounds, characterized by a strong antioxidant activity in different biological system.

## Figures and Tables

**Table 1:** Decorations on the hybrid structures **4a**–**k**, **5a**–**k** and the benzaldehydes **8a**–**m** used for their preparation.

Comp.	R	R_1_	R_2_	R_3_	R_4_
**4a, 8a**	H	O-CH_3_	O-CHF_2_	H	NH_2_
**4b, 8b**	O-cyclopentyl	O-CH_3_	H	H	NH_2_
**4c, 8c**	H	O-CH(CH_3_)CH_2_CH_3_	O-CH_3_	H	NH_2_
**4d, 8d**	H	O-cyclopentyl	O-CH_3_	H	NH_2_
**4e, 8e**	H	O-cyclopentyl	O-CHF_2_	H	NH_2_
**4f, 8f**	H	O-C_6_H_5_	O-CHF_2_	H	NH_2_
**4g, 8g**	H	O-CH_2_C_6_H_5_	O-CHF_2_	H	NH_2_
**4h, 8h**	H	O-CH_3_	O-CH_3_	O-CH_3_	NH_2_
**4i, 8i**	O-C_6_H_5_	O-CH_3_	H	H	NH_2_
**4j, 8j**	O-CH_2_C_6_H_5_	O-CH_3_	H	H	NH_2_
**4k, 8d**	H	O-cyclopentyl	O-CH_3_	H	1-pyrrolyl
**5a, 8k**	H	O-CH_3_	O-CH_3_	H	H
**5b, 8b**	O-cyclopentyl	O-CH_3_	H	H	H
**5c, 8c**	H	O-CH(CH_3_)CH_2_CH_3_	O-CH_3_	H	H
**5d, 8d**	H	O-cyclopentyl	O-CH_3_	H	H
**5e, 8e**	H	O-cyclopentyl	O-CHF_2_	H	H
**5f, 8l**	H	O-cyclohexyl	O-CH_3_	H	H
**5g, 8m**	H	O-CH_2_-O		H	H
**5h, 8h**	H	O-CH_3_	O-CH_3_	O-CH_3_	H
**5i, 8i**	O-C_6_H_5_	O-CH_3_	H	H	H
**5j, 8j**	O-CH_2_C_6_H_5_	O-CH_3_	H	H	H
**5k, 8d**	H	O-cyclopentyl	O-CH_3_		COCH_3_

**Table 2:** Inhibiting effect of compounds **4a**–**k** and **5a**–**j** on aggregation and reactive oxygen species (ROS) production expressed as IC_50_ (µΜ) values. Reported data are the mean +/- SD obtained in at least six different experiments each performed in duplicate.

Compd.	Aggregation Inhibition IC_50_ (µΜ)	ROS production Inhibition IC_50_ (µM)
**4a**	7.6 ± 0.5	10.1 ± 2.1
**4b**	64 ± 2.5	20.5 ± 2.5
**4c**	57.1 ± 1.6	16.7 ± 1.1
**4d**	29.8 ± 3.7	38.3 ± 2.8
**4e**	29.4 ± 4.4	32.8 ± 2.8
**4f**	9.8 ± 1.7	8.6 ± 2.2
**4g**	11.0 ± 1.2	9.5 ± 2.6
**4h**	>200	>200
**4i**	>200	>200
**4j**	>200	>200
**4k**	86.1 ± 7.8	92.7 ± 5.8
**5a**	31.5 ± 5.2	37.2 ± 2.5
**5b**	59.1 ± 5.5	66.7 ± 5.7
**5c**	70.9 ± 4.0	71.4 ± 6.0
**5d**	71.0 ± 6.8	70.6 ± 7.4
**5e**	70.5 ± 8.0	78.8 ± 5.6
**5f**	61.6 ± 6.5	59.9 ± 5.7
**5g**	67.0 ± 6.6	77.9 ± 7.1
**5h**	>200	>200
**5i**	>200	>200
**5j**	>200	>200
**5k**	>200	>200

## Supplementary Materials

The following are available online. Figure S1. 1H-NMR spectrum of compound 5a, Table S1: Elemental analysis of compounds 4a–k, 5a–k, 7, 9, 10, 12.

## Abbreviations

AD	Alzheimer’s Disease
COPD	Chronic obstructive pulmonary disease
fMLP	Formyl-methyl-leucyl-phenylalanine
IL-1	Interleukin 1
IL-8	Interleukine-8
MS	Multiple Sclerosis
p38 MAPK	p38 Mitogen-Activated Protein Kinase
PDE4	Phosphodiesterase type 4
PDE4Is	Phosphodiesterase type 4 Inhibitors
PDGF	Platelet derived growth factor
ROS	Reactive oxygen species
SAR	Structure-activity-relationship
TGF	Tumor growth factor
TNFα	Tumor necrosis factor alpha
