# Supplementary material for: New Hybrid Pyrazole and Imidazopyrazole Antinflammatory Agents Able to Reduce ROS Production in Different Biological Targets
Source: Molecules. 2020 Feb 18;25(4):899. doi: 10.3390/molecules25040899 (PMC7070443; doi:10.3390/molecules25040899)

Supplementary Information

New Hybrid Pyrazole and Imidazopyrazole Antinflammatory Agents Able to Reduce ROS Production in Different Biological Targets

Chiara Brullo [1,*], Matteo Massa [1], Federica Rapetti [1], Silvana Alfei [2], Maria B. Bertolotto [3], Fabrizio Montecucco [3], Maria Grazia Signorello [4] and Olga Bruno [1]

[1] Department of Pharmacy, Section of Medicinal Chemistry, University of Genoa, V.le Benedetto XV 3, I-16132 Genova, Italy; matteo_massa@outlook.it (M.M.), federica.rapetti@edu.unige.it (F.R.), obruno@unige.it (O.B.)

[2] Department of Pharmacy, Section of Chemistry and Pharmaceutical and Food Technologies, University of Genoa, Viale Cembrano 4, I-16148 Genova, Italy , alfei@difar.unige.it

[3] First Clinic of Internal Medicine, Department of Internal Medicine, and Centre of Excellence for Biomedical Research (CEBR), University of Genoa, Viale Benedetto XV 6, I-16132 Genoa Italy; Maria.Bianca.Bertolotto@unige.it (M.B.B.), Fabrizio.Montecucco@unige.it (F.M.)

[4] Department of Pharmacy, Biochemistry Lab., University of Genoa, Viale Benedetto XV 3, I-16132 Genova-Italy; Mariagrazia.Signorello@unige.it

* Correspondence: brullo@difar.unige.it; phone: 0039 010 3538368

Contents

Table S1. Elemental analysis of compounds **4a–k, 5a–k, 7, 9, 10, 12**

Figure S1. ^{1}H-NMR spectrum of compound **5a**

Table S1. Elemental analyses of compounds **4a–k, 5a–k, 7, 9, 10, 12**

Comp.	Molecular formula	MW		%C	%H	%N
**4a**	$C_{21}H_{21}F_2N_5O_4$	445.42	Calculated	56.63	4.75	15.72
			Experimental	56.63	4.86	15.56
**4b**	$C_{25}H_{29}N_5O_4$	463.53	Calculated	64.78	6.31	15.11
			Experimental	64.41	6.69	15.26
**4c**	$C_{24}H_{29}N_5O_4$	451.52	Calculated	63.84	6.47	15.51
			Experimental	63.92	6.81	15.63
**4d**	$C_{25}H_{29}N_5O_4$	463.53	Calculated	64.78	6.31	15.11
			Experimental	64.65	6.69	15.04
**4e**	$C_{25}H_{27}F_2N_5O_4$	499.51	Calculated	60.11	5.45	14.02
			Experimental	60.10	5.38	14.17
**4f**	$C_{26}H_{23}F_2N_5O_4$	507.49	Calculated	61.53	4.57	13.80
			Experimental	61.59	4.58	13.31
**4g**	$C_{27}H_{25}F_2N_5O_4$	521.52	Calculated	62.18	4.83	13.43
			Experimental	62.20	4.88	13.41
**4h**	$C_{22}H_{24}N_4O_5$	424.45	Calculated	60.13	5.73	15.94
			Experimental	60.12	5.76	15.79
**4i**	$C_{26}H_{25}N_5O_4$	471.51	Calculated	66.23	5.34	14.85
			Experimental	66.16	5.59	14.72
**4j**	$C_{27}H_{27}N_5O_4$	485.53	Calculated	66.79	5.61	14.42
			Experimental	66.88	5.76	14.10
**4k**	$C_{29}H_{31}N_5O_4$	513.59	Calculated	67.82	6.08	13.64
			Experimental	67.39	6.20	13.96

Table S1. *Cont.*

**5a**	$C_{21}H_{21}N_5O_3$	391.42	Calculated	64.44	5.41	17.89
			Experimental	64.07	6.15	17.55
**5b**	$C_{25}H_{27}N_5O_3$	445.51	Calculated	67.40	6.11	15.72
			Experimental	67.49	6.13	15.34
**5c**	$C_{24}H_{27}N_5O_3$	433.50	Calculated	66.49	6.28	16.16
			Experimental	67.49	6.13	15.34
**5d**	$C_{25}H_{27}N_5O_3$	445.51	Calculated	67.40	6.11	15.72
			Experimental	67.41	6.10	15.35
**5e**	$C_{25}H_{25}F_2N_5O_3$	481.49	Calculated	62.36	5.23	14.55
			Experimental	62.10	5.60	14.32
**5f**	$C_{26}H_{29}N_5O_3$	459.54	Calculated	67.95	6.36	15.24
			Experimental	67.62	6.38	15.59
**5g**	$C_{20}H_{17}N_5O_3$	375.38	Calculated	63.99	4.56	18.66
			Experimental	63.65	4.87	18.83
**5h**	$C_{22}H_{23}N_5O_4$	421.45	Calculated	62.70	5.50	16.62
			Experimental	62.96	5.43	16.31
**5i**	$C_{26}H_{23}N_5O_3$	453.49	Calculated	68.86	5.11	15.44
			Experimental	68.68	5.24	15.26
**5j**	$C_{27}H_{25}N_5O_3$	467.52	Calculated	69.36	5.39	14.98
			Experimental	69.27	5.74	15.26
**5k**	$C_{27}H_{28}N_5O_4$	487.55	Calculated	66.51	6.00	14.36
			Experimental	66.71	6.40	14.24
**7**	$C_{12}H_{15}N_5O_2 1/2H_2O$	540.57	Calculated	53.52	5.61	26.01
			Experimental	53.36	5.66	26.35
**9**	$C_{18}H_{19}N_3O_3$	325.36	Calculated	66.45	5.89	12.91
			Experimental	66.87	5.99	12.71
**10**	$C_{16}H_{17}N_5O_2$	311.34	Calculated	61.72	5.50	22.49
			Experimental	61.97	5.57	22.19
**12**	$C_{12}H_{13}N_5O$	243.26	Calculated	59.25	5.39	28.79
			Experimental	59.02	5.44	28.60

Figure S1. ^{1}H-NMR spectrum of **5a**

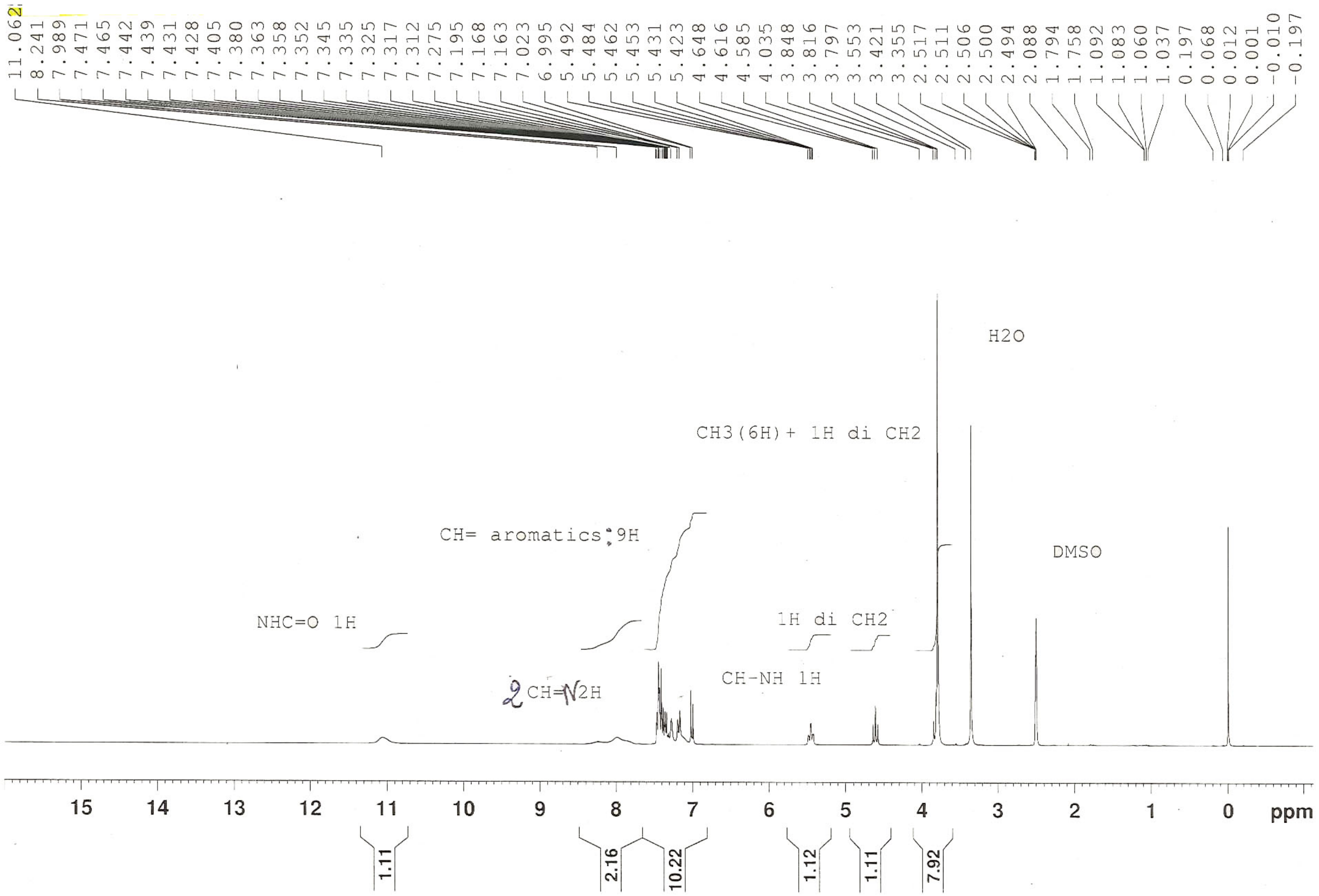

Supplement: Supplementary file 1 [file molecules-25-00899-s001.pdf]
